# Comprehensive Assessment of Biventricular and Biatrial Myocardial Strain Parameters at Six Years Postpartum in a Cohort of Women with Previous Hypertensive Disorders of Pregnancy

**DOI:** 10.3390/jcm14134767

**Published:** 2025-07-05

**Authors:** Andrea Sonaglioni, Federico Napoli, Rebecca Dell’Anna, Gian Luigi Nicolosi, Stefano Bianchi, Michele Lombardo, Sergio Harari, Chiara Lonati

**Affiliations:** 1 Division of Cardiology, IRCCS MultiMedica, 20138 Milan, Italy; michele.lombardo@multimedica.it; 2 Division of Internal Medicine, IRCCS MultiMedica, 20138 Milan, Italy; federico.napoli@multimedica.it (F.N.); sergio@sergioharari.it (S.H.); chiara.lonati@multimedica.it (C.L.); 3 Division of Gynaecology and Obstetrics, IRCCS MultiMedica, 20138 Milan, Italy; rebecca.dellanna@studenti.unimi.it (R.D.); stefano.bianchi@unimi.it (S.B.); 4 Division of Cardiology, Policlinico San Giorgio, 33170 Pordenone, Italy; gianluigi.nicolosi@gmail.com; 5 Department of Clinical Sciences and Community Health, Università Di Milano, 20126 Milan, Italy

**Keywords:** previous hypertensive disorders of pregnancy, previous pre-eclampsia, speckle tracking echocardiography, subclinical myocardial dysfunction, subclinical carotid atherosclerosis

## Abstract

**Background:** Over the past decade, few echocardiographic investigations have assessed myocardial strain parameters in women with a history of hypertensive disorders of pregnancy (HDP), and their findings have been inconsistent. Moreover, no study has comprehensively evaluated deformation indices of all biventricular and biatrial chambers in women post-HDP. This study aimed to examine the structural and functional myocardial properties of all cardiac chambers in a cohort of women with prior HDP at six years after delivery. **Methods:** We analyzed a consecutive cohort of women with previous HDP and compared them with a control group of normotensive healthy women matched for age and body mass index (BMI). Both groups underwent standard transthoracic echocardiography (TTE) supplemented by a detailed speckle tracking echocardiography (STE) evaluation of biventricular and biatrial myocardial deformation, along with carotid ultrasound, at six years postpartum. The primary endpoint was subclinical myocardial dysfunction, defined by impaired left ventricular global longitudinal strain (LV-GLS < 20%), while the secondary endpoint was early carotid atherosclerosis, defined by common carotid artery intima-media thickness (CCA-IMT) ≥ 0.7 mm. **Results:** The study included 31 women with previous HDP (mean age 42.3 ± 5.9 years) and 30 matched controls without HDP history (mean age 40.8 ± 5.0 years). The average follow-up duration was 6.1 ± 1.3 years postpartum. Despite preserved and comparable systolic function on conventional TTE, most myocardial strain and strain rate measures in both ventricles and atria were significantly reduced in the HDP group compared to controls. Subclinical myocardial dysfunction was detected in 58.1% of women with prior HDP, and 67.7% exhibited increased CCA-IMT (≥0.7 mm). A history of pre-eclampsia (PE) was independently associated with subclinical myocardial dysfunction (HR 4.01, 95% CI 1.05–15.3, *p* = 0.03). Both third-trimester BMI (HR 1.21, 95% CI 1.07–1.38, *p* = 0.003) and PE (HR 6.38, 95% CI 1.50–27.2, *p* = 0.01) independently predicted early carotid atherosclerosis. Notably, a third-trimester BMI above 27 kg/m^2^ showed optimal sensitivity and specificity for identifying the secondary outcome. **Conclusions:** A history of PE is independently associated with a higher risk of subclinical myocardial dysfunction and early carotid atherosclerosis at six years postpartum.

## 1. Introduction

Hypertensive disorders of pregnancy (HDP) are relatively frequent, affecting up to 10% of pregnancies globally [1]. According to major guidelines [2,3], HDP encompasses the following: (1) Chronic hypertension, defined as arterial hypertension present before pregnancy or prior to 20 weeks of gestation or the use of antihypertensive medication before pregnancy; (2) Gestational hypertension (GH), characterized by elevated blood pressure arising after 20 weeks of gestation without significant proteinuria; (3) Pre-eclampsia (PE), which involves new-onset hypertension after 20 weeks, accompanied by proteinuria and/or maternal organ dysfunction such as acute kidney injury, hepatic impairment, neurological symptoms, hemolysis, thrombocytopenia, or fetal growth restriction; and (4) Chronic hypertension complicated by superimposed GH with proteinuria.

Postpartum, women with a history of HDP exhibit alterations in cardiac structure and function that predispose them to an increased risk of long-term cardiovascular (CV) complications, including myocardial infarction, heart failure, stroke, and CV-related mortality [4,5,6]. Evidence from the literature indicates that the initial decade after delivery represents a particularly high-risk period for the development of CV events [7,8,9,10,11], suggesting that women with previous HDP (pHDP) may experience adverse cardiac outcomes at a relatively young age. Early identification of cardiac structural and functional abnormalities in this population is therefore critical to prevent progression to clinical disease.

Recent advances in cardiac imaging have introduced speckle tracking echocardiography (STE), a technique capable of detecting subclinical myocardial dysfunction at an early stage [12]. The left ventricular (LV) global longitudinal strain (GLS), the most commonly utilized STE-derived metric of myocardial contractility, identifies systolic impairment earlier than conventional left ventricular ejection fraction (LVEF) measured by transthoracic echocardiography (TTE), facilitating the recognition of subclinical myocardial damage [13].

To date, only a limited number of studies have investigated LV-GLS via STE in women with prior HDP, with findings remaining inconclusive [14,15,16,17,18]. Moreover, no comprehensive assessment encompassing deformation indices of all biventricular and biatrial chambers has been reported in this population.

Given the elevated CV risk observed in women with pHDP during the first decade postpartum [7,8,9,10,11], this study was designed to thoroughly evaluate the structural and deformation characteristics of all cardiac chambers in a cohort of women with pHDP, compared to a control group of healthy women with previous uncomplicated pregnancies, at six years postpartum.

## 2. Materials and Methods

### 2.1. Patient Selection

This case–control study evaluated a consecutive cohort of women with a history of hypertensive disorders of pregnancy in comparison to an age- and body mass index (BMI)-matched control group of normotensive women with prior uncomplicated pregnancies, conducted between February and April 2024. Both groups delivered at the Department of Gynecology and Obstetrics of San Giuseppe Multimedica IRCCS Hospital (Milan) between February 2017 and May 2018. Approximately one-third of the pHDP participants had previously been included in an earlier study assessing left atrial reservoir strain (LASr) in pregnant women with GH [19].

Inclusion criteria comprised women with a documented history of GH, defined as newly diagnosed hypertension arising after 20 weeks’ gestation or within 48 h postpartum [20], and/or PE, characterized by GH accompanied by new-onset proteinuria (≥0.3 g per 24-h urine collection) [21]. Exclusion criteria included preexisting hypertension or diabetes mellitus, gestational diabetes mellitus, significant comorbid conditions (such as cardiovascular, respiratory, or renal diseases), hemodynamic instability, and inadequate echocardiographic acoustic windows that precluded proper delineation of ventricular and atrial endocardial borders.

Hypertension was defined according to standard criteria as a sustained systolic blood pressure (SBP) ≥ 140 mmHg or diastolic blood pressure (DBP) ≥ 90 mmHg [22]. At each clinical visit, blood pressure was measured three times at two-minute intervals on the same arm, after the participant had been seated at rest for at least five minutes; only the third measurement was recorded. Additionally, participants underwent electrocardiography (ECG), conventional TTE with comprehensive STE analysis of both ventricles and atria, and carotid ultrasonography. All imaging assessments were performed on the same day by a single cardiologist (A.S.) blinded to clinical data.

The study adhered to the ethical principles outlined in the Declaration of Helsinki and was approved by the local Ethics Committee (reference number 506/24). Written informed consent was obtained from all participants prior to enrollment.

### 2.2. Clinical and Instrumental Parameters

Table 1 lists all the clinical and instrumental parameters collected in the two cohorts of women included in the present study and the methods employed for their assessment.

### 2.3. Statistical Analysis

The primary aim of this study was to quantitatively evaluate biventricular and biatrial myocardial function using STE in women with a history of hypertensive disorders of pregnancy, and to compare these findings with those of age- and BMI-matched control subjects with prior normotensive pregnancies, assessed at six years postpartum. The secondary aim was to compare common carotid artery intima-media thickness (CCA-IMT) between the two groups over the follow-up period.

A priori sample size estimation indicated that recruiting 30 women with prior HDP and 30 matched healthy controls would yield 80% power to detect a difference of two percentage points in GLS values (20% vs. 18%) at six years postpartum. This calculation assumed a standard deviation of 2.5 per group, applying a two-sided equal-variance *t*-test with a significance threshold of 0.05.

Continuous variables were tested for normality using the Kolmogorov–Smirnov test. Variables conforming to a normal distribution are reported as mean ± standard deviation and compared via independent two-tailed *t*-tests. Non-normally distributed data are presented as median with range and analyzed using the Mann–Whitney U test. Categorical variables were compared using chi-square tests.

Cox proportional hazards regression models were utilized to identify independent predictors of subclinical myocardial dysfunction—defined as an absolute LV-GLS value below 20% in the context of preserved LVEF (≥55%) [38]—and subclinical carotid atherosclerosis, defined as CCA-IMT ≥ 0.7 mm [45], among women with prior HDP during follow-up. Following the “one predictor per ten events” guideline, variables entered into the Cox models included third-trimester age (as demographic index), third-trimester BMI (as anthropometric index), and history of PE (as hypertensive disorder of pregnancy) for both outcomes; chronic antihypertensive treatment (as index of the current medical treatment) was included for the primary outcome only and current high-density lipoprotein (HDL) cholesterol levels (as metabolic index) for the secondary outcome only.

Receiver operating characteristic (ROC) curve analysis was conducted to determine the sensitivity and specificity of the principal statistically significant continuous predictor for the secondary outcome over follow-up, with area under the curve (AUC) calculated accordingly.

Intra-observer and inter-observer reproducibility of LV-GLS measurements via STE were evaluated in a randomly selected subgroup of 15 women with prior HDP. Measurements were repeated independently by the initial examiner (A.S.) and a second cardiologist (M.L.), both blinded to previous results. Reliability was assessed using the intraclass correlation coefficient (ICC) with 95% confidence intervals, with ICC values ≥ 0.70 indicating satisfactory agreement.

All statistical analyses were performed using SPSS version 28 (IBM Corp., Chicago, IL, USA). A two-tailed *p*-value of less than 0.05 was considered statistically significant.

## 3. Results

### 3.1. Clinical Findings

A total of 31 women with a history of HDP and 30 healthy controls, matched for age and BMI, were assessed at six years postpartum.

The principal clinical, obstetric, hemodynamic, and laboratory parameters collected during the third trimester of pregnancy in both cohorts are comprehensively summarized in Table 2.

The majority of women in both groups were Caucasian and aged 35 years or older. More than half of the women with previous HDP (58.1%) reported a family history of hypertension, while about one-third (32.3%) had dyslipidemia and approximately two-thirds (61.3%) experienced PE during pregnancy. All PE cases were diagnosed at or beyond 34 weeks of gestation. Compared to the control group, pHDP women delivered significantly earlier. Most pHDP women (87.1%) received antihypertensive treatment during pregnancy, predominantly calcium channel blockers and alpha-2 agonists, with alpha-beta blockers prescribed less frequently.

At six years postpartum, both groups exhibited a low incidence of smoking and type 2 diabetes. However, dyslipidemia and obesity were more common among pHDP women than in controls. Within the pHDP group, 10 women (32.3%) were regularly taking antihypertensive medication and 11 (35.5%) had blood pressure readings of ≥140/90 mmHg during clinical evaluation. Among those on antihypertensive therapy, three (30%) still had uncontrolled hypertension at the visit. Additionally, of the pHDP women with elevated blood pressure (≥140/90 mmHg), eight (72.7%) were not receiving antihypertensive treatment (Table 3).

### 3.2. Instrumental Findings

Table 4 presents the morphological, functional, and hemodynamic variables measured by conventional TTE and carotid ultrasonography in both groups of women at six years postpartum.

Transthoracic echocardiography revealed comparable biventricular and biatrial chamber dimensions between the two groups. Despite the absence of overt pathological LV remodeling, women with prior HDP exhibited significantly increased relative wall thickness (RWT) and left ventricular mass index (LVMi) compared to controls. LV systolic function, evaluated by LVEF, remained within normal limits in both cohorts. Assessment of LV diastolic function demonstrated a significantly reduced E/A ratio alongside an elevated E/average e’ ratio in the pHDP group relative to controls. No significant valvular abnormalities were observed in either group. Pulmonary hemodynamic evaluation indicated significantly lower tricuspid annular plane systolic excursion (TAPSE) and TAPSE/systolic pulmonary artery pressure (sPAP) ratios in women with previous HDP versus controls. Furthermore, the aortic root diameter was significantly enlarged in the pHDP cohort.

Hemodynamic analysis showed a significantly reduced stroke volume (SV) in pHDP women compared to controls, while heart rate and cardiac output (CO) were similar between groups. Total peripheral resistance index (TPRi) was significantly elevated in the pHDP group.

Regarding ventricular–arterial coupling (VAC) parameters, women with pHDP demonstrated significantly higher arterial elastance indexes (EaIs) compared to controls, whereas end-systolic elastance index (EesI) was comparable between groups. Consequently, the VAC ratio (EaI/EesI) did not differ significantly.

Carotid ultrasonography revealed that mean values of common carotid artery intima-media thickness (CCA-IMT), relative wall thickness (CCA-RWT), and cross-sectional area (CCA-CSA) were all significantly increased in pHDP women compared to controls.

Speckle tracking echocardiography demonstrated that most biventricular and biatrial myocardial strain and strain rate parameters were significantly impaired in the pHDP group. Specifically, absolute values of LV-GLS, right ventricular GLS (RV-GLS), LASr, and right atrial strain during reservoir phase (RASr) were significantly lower in pHDP women versus controls, whereas LV global circumferential strain (GCS) was similar between the groups. The reduction in LASr and RASr was predominantly driven by decreased conduit longitudinal strain in the left and right atria, while atrial contractile strain was preserved in both cohorts. Overall, more than 50% of the women with prior HDP exhibited reduced biventricular and biatrial myocardial strain parameters relative to established reference values [38,39,40,41,42]. Notably, approximately 20% of the healthy controls also demonstrated mild attenuation in myocardial deformation indices (Table 5).

**Figure 1:**
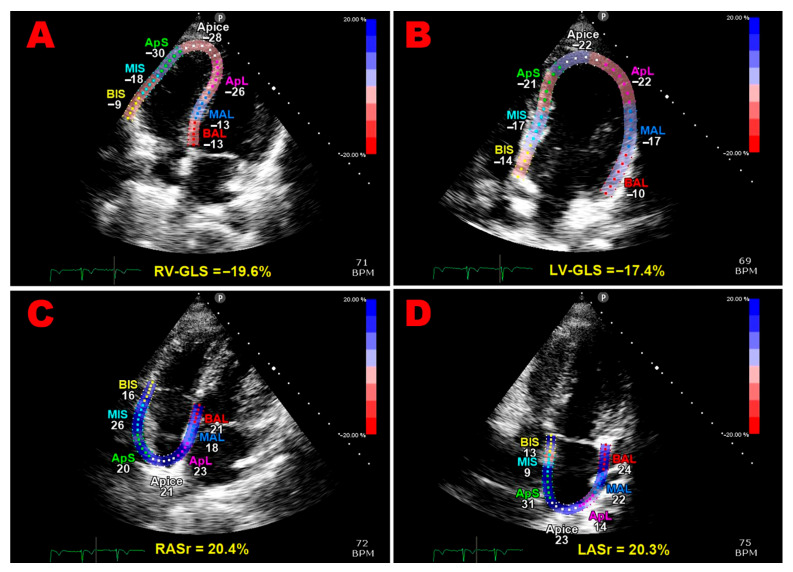
Illustrations of right ventricular global longitudinal strain (**A**), left ventricular global longitudinal strain (**B**), right atrial reservoir strain (**C**), and left atrial reservoir strain (**D**) measurements obtained from the apical four-chamber view in a pHDP patient from this study. All myocardial strain values are decreased relative to the established reference ranges. GLS, global longitudinal strain; LASr, left atrial reservoir strain; LV, left ventricular; pHDP, previous hypertensive disorder of pregnancy; RASr, right atrial reservoir strain; RV, right ventricular.

Multipanel Figure 1 illustrates examples of biventricular and biatrial longitudinal strain parameters measured from the apical four-chamber view in a pHDP woman included in the present study.

### 3.3. Follow-Up Data

The average follow-up duration after delivery was 6.1 ± 1.3 years. Throughout this period, none of the women with a history of HDP exhibited clinical signs or symptoms of cardiomyopathy, and no major adverse CV events were documented. Nonetheless, subclinical myocardial dysfunction detected by STE was present in more than half of the pHDP cohort (58.1%), alongside persistent arterial hypertension, at six years postpartum. Specifically, nine pHDP women (29%) exhibited both sustained impairment in LV-GLS and chronic hypertension during follow-up. Additionally, subclinical carotid atherosclerosis was identified in 21 pHDP participants (67.7%).

Cox regression analysis aimed at identifying independent predictors of subclinical myocardial dysfunction—defined as LV-GLS less than 20% [38] at six years postpartum—revealed that only a history of PE (HR 4.01; 95% CI], 1.05–15.3; *p* = 0.03) was independently associated with the primary outcome (Table 6).

**Figure 2:**
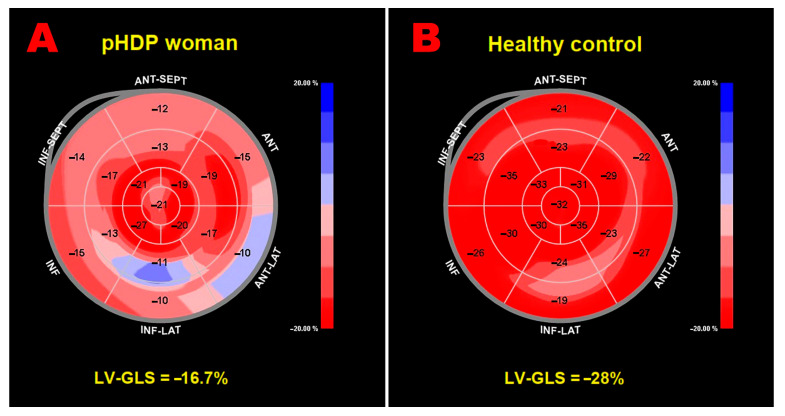
Examples of LV-GLS bull’s-eye plots obtained in a pHDP woman with a history of pregnancy complicated by obesity and PE (**A**) and in a woman with previous uncomplicated pregnancy (**B**), respectively. GLS, global longitudinal strain; LV, left ventricular; PE, pre-eclampsia; pHDP, previous hypertensive disorder of pregnancy.

Figure 2 displays representative LV-GLS bull’s-eye plots from a pHDP woman with a third-trimester BMI exceeding 27 kg/m^2^ and a pregnancy complicated by PE (Panel A) and from a woman with a history of an uncomplicated pregnancy (Panel B).

Cox regression analysis to identify independent predictors of subclinical carotid atherosclerosis at the six-year follow-up revealed that third-trimester BMI (HR 1.21, 95% CI 1.07–1.38, *p* = 0.003) and PE (HR 6.38, 95% CI 1.50–27.2, *p* = 0.01) were significantly and independently linked to the secondary outcome (Table 7).

**Figure 3:**
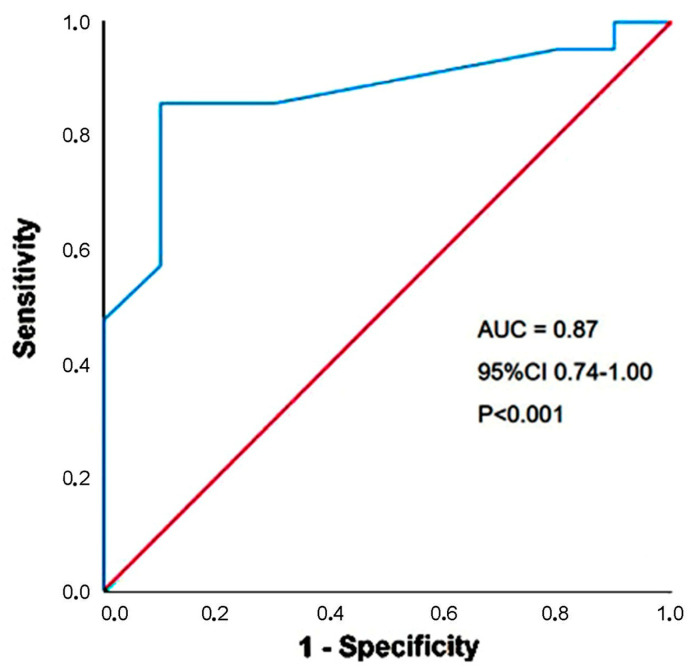
ROC curve analysis conducted to determine the sensitivity and specificity of third-trimester BMI in predicting the secondary outcome throughout the follow-up period. AUC, area under curve; BMI, body mass index; ROC, receiver operating characteristics.

A third-trimester BMI > 27 kg/m^2^ had 86% sensitivity and 90% specificity (AUC = 0.87; 95% CI 0.74–1.00, *p* = 0.004) for predicting the secondary outcome (Figure 3).

### 3.4. Measurement Variability

An in-depth evaluation of both intra- and inter-observer variability for LV-GLS measurements was performed on a randomly selected subgroup of 15 women with prior HDP. The ICCs indicated excellent agreement, with intra-observer reliability at 0.97 (95% CI: 0.91–0.99) and inter-observer reliability at 0.92 (95% CI: 0.78–0.97).

## 4. Discussion

### 4.1. Main Findings of the Present Study

This case–control study revealed that women with a history of HDP, compared to healthy women with prior uncomplicated pregnancies, exhibited the following: (1) subtle LV remodeling marked by increased RWT and LVMi without signs of concentric remodeling or hypertrophy; (2) a modest decrease in the E/A ratio alongside a corresponding rise in the E/average e’ ratio, yet without evidence of elevated LV filling pressures; (3) reduced TAPSE and TAPSE/sPAP ratio, though no pathological right ventricular–pulmonary artery (RV-PA) uncoupling (defined as TAPSE/sPAP < 0.80) [27]; (4) elevated arterial elastance values, albeit remaining within normal limits [46]; and (5) early carotid artery structural changes demonstrated by increased CCA-IMT, CCA-RWT, and CCA-CSA.

Although conventional TTE showed preserved LVEF, 2D-STE detected significant impairments in multiple biventricular and biatrial myocardial strain parameters in the pHDP group compared to controls. No major cardiovascular events occurred during the six-year postpartum follow-up. However, more than half of the pHDP women exhibited subclinical myocardial dysfunction as assessed by strain imaging and displayed early signs of carotid atherosclerosis at six years postpartum.

In our findings, PE was independently associated with a 4-fold and 6-fold increased risk of developing subclinical myocardial dysfunction and early carotid atherosclerosis, respectively, at six years postpartum. Additionally, a history of being overweight or having obesity during pregnancy, indicated by a third-trimester BMI exceeding 27 kg/m^2^, independently predicted the secondary outcome.

### 4.2. Comparison with Previous Studies and Interpretation of Results

Our results align with prior research involving women with a history of hypertensive disorders of pregnancy, which consistently demonstrated early abnormalities in LV-GLS identified through STE. For instance, Clemmensen et al. [14] reported that individuals with early-onset PE exhibited a higher likelihood of subclinical LV dysfunction 12 years after pregnancy, with LV-GLS showing the strongest association among assessed cardiac parameters. Similarly, Boardman et al. [15] studied 103 pHDP participants and 70 normotensive controls 5 to 10 years postpartum, finding that the pHDP group displayed altered cardiac structure characterized by increased LVMi and left atrial volume index (LAVi), as well as reduced E/A ratio and LV-GLS. In another study, Levine et al. [16] observed that pHDP women who later developed hypertension demonstrated more pronounced LV remodeling, such as elevated RWT, impaired diastolic function, reduced LV-GLS, and increased arterial elastance, compared to those who remained normotensive, attributing these changes primarily to subsequent hypertension. Additionally, Gronningsaeter L. [17] found persistent hypertension, increased LV mass, and diminished systolic and diastolic performance seven years after severe PE. In contrast, Al-Nashi et al. [18], in a small-scale study of women 11 years after PE, reported no long-term differences in cardiac function or ventricular–arterial interaction when compared to a control group with normotensive pregnancies.

Unlike previous studies that focused solely on LV-GLS, our investigation evaluated myocardial deformation across all four heart chambers in pHDP women. We observed subclinical dysfunction affecting both ventricles and atria, while circumferential LV strain remained intact, likely a compensatory mechanism preserving overall systolic function. This aligns with established findings that endocardial (longitudinal) strain reductions precede mid-wall (circumferential) changes [47].

Our findings reinforce the superiority of STE over conventional TTE for detecting subtle myocardial impairments, even in the absence of symptoms and with preserved LVEF (≥55%) [12].

Pathophysiologically, the attenuated strain values in pHDP women may result from chronically elevated afterload, a known driver of myocardial remodeling and early diastolic dysfunction [48,49]. Increased RV afterload, reflected in reduced TAPSE/sPAP ratio, may similarly affect right heart mechanics [50].

Metabolic comorbidities, such as obesity, dyslipidemia, insulin resistance, and hyperinsulinemia, likely exacerbate myocardial fibrosis and stiffness, further reducing biventricular and atrial strain [51,52]. Additional factors such as chest wall morphology or increased thoracic adiposity may also mechanically limit cardiac deformation, though this was not evaluated in our study [53,54]. Anthropometric factors, especially a chest wall that is concave in shape or conditions like pectus excavatum, may have played a role in the reduced myocardial strain observed in about 20% of healthy individuals with prior uncomplicated pregnancies. In fact, our earlier work showed that a reduced front-to-back (anteroposterior) chest diameter can lead to lower basal longitudinal strain, likely due to pressure from the sternum, even in individuals without clear signs of heart disease, such as those diagnosed with mitral valve prolapse [55].

In line with earlier research [56,57,58,59,60,61], our findings revealed that women with a history of hypertensive disorders of pregnancy exhibited a higher burden of atherosclerosis, indicated by increased CCA-IMT, CCA-RWT, and CCA-CSA, six years after childbirth. Several underlying pathophysiological mechanisms may account for the link between PE and the early development of carotid atherosclerosis, particularly within the first 10 years post-delivery. One explanation is that HDP and CV disease share a set of risk factors and represent different clinical manifestations of the same underlying condition at various life stages. This theory is supported by strong correlations between HDP and classic CV risk factors such as chronic hypertension, type 2 diabetes, dyslipidemia, and elevated BMI, as previously reported [62]. Additionally, the association between HDP and higher levels of blood pressure, BMI, and lipids later in life may be significantly influenced by CV risk factors present before pregnancy [63]. Placental abnormalities often found in cases of PE resemble early-stage atherosclerotic changes [64], possibly indicating a predisposition to long-term vascular dysfunction [65]. Moreover, subclinical carotid atherosclerosis may also stem from persistent endothelial dysfunction initiated by HDP, as evidenced by markers of endothelial damage [66] and systemic inflammation [67] detectable up to eight years after affected pregnancies.

Finally, our results echo large-scale epidemiological data linking PE and GH to elevated CV risks: Kestenbaum et al. reported a 2.2–3.3-fold increase in CV hospitalizations over approximately eight years [8]; Cain et al. documented a 42% higher risk of CV disease within five years [9]; Egeland et al. found a 6–7-fold rise in treated hypertension over 10 years [10]; Jarvie et al. noted ~2.4-fold greater odds of early CV hospitalization, especially among African American women [11]; and Levine et al. observed a 2.4-fold increased risk of hypertension a decade after HDP [16].

### 4.3. Implications for Clinical Practice

Given the established link between hypertensive disorders of pregnancy, particularly PE, and the heightened risk of subclinical myocardial dysfunction and early carotid atherosclerosis within the first decade postpartum, current guidelines and expert consensus advocate for systematic CV risk screening and the implementation of preventive strategies in this population [68].

Cardiovascular disease prevention in pHDP women should commence early after childbirth and be sustained throughout life. This approach includes an initial postpartum evaluation, risk factor assessment, and a multidisciplinary intervention focusing on lifestyle modifications at 6–12 weeks and again at one year postpartum, followed by routine annual evaluations and a comprehensive review at midlife (around age 50) [69].

Based on our study’s findings, the use of strain echocardiography warrants consideration for integration into standard care, especially during pregnancies affected by PE and within the first 10 years postpartum. This advanced imaging technique offers additional diagnostic and prognostic insights into both ventricular and atrial myocardial mechanics, complementing conventional TTE. Detection of subtle impairments in myocardial strain or early carotid artery remodeling in pHDP women may prompt clinicians to initiate or intensify pharmacologic therapies and lifestyle interventions, such as calorie-restricted diets and weight management, with the goal of reducing long-term CV risk.

### 4.4. Limitations of the Study

This study has several limitations. Firstly, its single-center design and relatively small sample size of women with prior HDP may limit generalizability; however, the sample size was appropriately determined through a rigorous power analysis. Additionally, participants did not undergo baseline echocardiographic assessments prior to pregnancy, preventing a definitive conclusion on whether the observed impairments in biventricular and biatrial deformation parameters predated the onset of GH and/or PE. Furthermore, data on lifestyle factors such as diet, physical activity, and hormonal status, each of which could influence cardiovascular remodeling, were not collected at enrollment. Another limitation is that myocardial strain analysis was performed using a single software platform, originally designed for LV-GLS, which was the only available tool at our institution. It is important to acknowledge that strain echocardiography has inherent technical constraints, including dependency on optimal image quality, sufficient frame rates (typically ≥ 40 fps), operator expertise, hemodynamic conditions, the ultrasound equipment used, and chest wall anatomy [70,71,72,73]. Lastly, no blood tests were included in the study protocol to assess markers such as C-reactive protein, NT-proBNP, or HOMA-IR. The absence of these inflammatory, metabolic, and hemodynamic indicators limited our ability to further elucidate the mechanisms underlying the cardiac and carotid changes observed in pHDP women at six years postpartum.

## 5. Conclusions

A prior diagnosis of PE is independently linked to a higher likelihood of developing subclinical myocardial dysfunction and early carotid atherosclerosis at six years postpartum.

Incorporating strain echocardiographic imaging into routine clinical practice could help detect subclinical myocardial dysfunction early in women with a history of hypertensive disorders of pregnancy. This would allow for timely intervention, such as intensified antihypertensive therapy and closer monitoring, to lower the risk of future CV complications.

Further prospective, multicentric studies with a larger cohort of pHDP women are necessary to validate our findings and support their translation into routine clinical practice.

## Figures and Tables

**Table 1:** Demographic, anthropometric, obstetrical, clinical, hemodynamic, conventional echocardiographic, myocardial strain and carotid ultrasound parameters measured in pHDP women and controls.

**Demographic, anthropometric, obstetrical, and clinical parameters**	Age, ethnicity, BSA, BMI, parity, gestational week of hypertension onset, gestational age at delivery, prevalence of smoking, dyslipidemia and family history of hypertension, relevant comorbidities, electrocardiographic data, serum levels of haemoglobin, creatinine and eGFR [23], fasting glucose, lipid profile and uric acid, previous evidence of proteinuria, and both the previous and the current antihypertensive therapy.
**Conventional echoDoppler parameters by using Philips Sparq ultrasound machine (Philips, Andover, MA, USA) with a 2.5 MHz transducer**	Aortic root and ascending aorta; RWT = 2PWT/LVEDD; LVMi; LVEF [24]; LAVi; RVIT; TAPSE; E/A ratio; E/average e’ ratio [25]; sPAP = 4TRV^2^ + RAP [26]; TAPSE/sPAP ratio [27].
**Hemodynamic indices**	Brachial SBP and DBP; MAP = DBP + [(SBP-DBP/3)] [28]; PP = SBP-DBP [29]; SV = LVOT area X LVOT VTI [30]; CO = SV X HR [30]; TPR = MAP/CO X 80 [31]; ESP = 0.9 X SBP [32]; EaI = ESP/SVindex ratio [33]; EesI = ESP/LVESVi [33]; VAC = EaI/EesI ratio [33].
**Myocardial strain parameters** **by using Philips QLAB 10.3.1 ultrasound software and Q-Analysis module [34].**	LV-GLS; LV-GCS [34]; RV-GLS; RV-FWLS [35]; LASr = LAScd + LASct (biplane method); LA-GSR+, LA-GSRE and LA-GSRL [36]; LA stiffness = LASr/E/average e’ ratio [37]; RASr = RAScd + RASct; RA-GSR+, RA-GSRE and RA-GSRL. Absolute values inferior to 20% for LV-GLS [38], 23.3% for LV-GCS [39], 20% for RV-GLS [40], 39% for LASr [41], and 35% for RASr [42] were considered to be abnormal.
**Carotid ultrasound parameters** **by using Philips Sparq ultrasound machine with a 12 MHz transducer.**	Av. left and right CCA-IMT; av. left and right CCA-EDD; av. left and right carotid RWT = 2 × average IMT/average CCA-EDD; av. left and right CCA-CSA = [π × (2 × average IMT + average CCA-EDD)/2)^2^ − π × (average CCA-EDD/2)^2^] [43]. Based on the accepted reference ranges for age and sex [44,45], CCA-IMT values ≥ 0.7 mm were considered to be abnormal.

BMI, body mass index; BSA, body surface area; CCA, common carotid artery; CO, cardiac output; CSA, cross-sectional area; DBP, diastolic blood pressure; EaI, arterial elastance index; EDD, end-diastolic diameter; EesI, end-systolic elastance index; eGFR, estimated glomerular filtration rate; ESP, end-systolic pressure; FWLS, free wall longitudinal strain; GCS, global circumferential strain; GLS, global longitudinal strain; GSR+, positive global strain rate; GSRE, global early-diastolic strain rate; GSRL, global late-diastolic strain rate; HR, heart rate; IMT, intima-media thickness; LA, left atrial; LAScd, left atrial conduit strain; LASct, left atrial contractile strain; LASr, left atrial reservoir strain; LAVi, left atrial volume index; LV, left ventricular; LVEDD, left ventricular end-diastolic diameter; LVEF, left ventricular ejection fraction; LVOT, left ventricular outflow tract; LVMi, left ventricular mass index; MAP, mean arterial pressure; PWT, posterior wall thickness; RA, right atrial; RAP, right atrial pressure; RAScd, right atrial conduit strain; RASct, right atrial contractile strain; RASr, right atrial reservoir strain; RV, right ventricular; RVIT, right ventricular inflow tract; RWT, relative wall thickness; SBP, systolic blood pressure; sPAP, systolic pulmonary artery pressure; SV, stroke volume; TAPSE, tricuspid annular plane systolic excursion; TPR, total peripheral resistance; TRV, tricuspid regurgitation velocity; VAC, ventricular-arterial coupling; VTI, velocity time integral.

**Table 2:** Clinical, obstetrical, hemodynamic, and laboratory parameters collected in HDP women and controls at the third trimester of pregnancy.

	HDP Women (n = 31)	Controls (n = 30)	*p*-Value
**Demographics, anthropometrics, cardiovascular risk factors, and obstetrics**			
Age (yrs)	36.6 ± 5.9	36.7 ± 2.9	0.93
Age ≥ 35 yrs (%)	22 (70.9)	20 (66.6)	0.72
Caucasian ethnicity (%)	27 (87.1)	26 (86.7)	0.96
BSA (m^2^)	1.87 ± 0.16	1.85 ± 0.11	0.57
BMI (kg/m^2^)	28.0 ± 4.7	27.5 ± 2.8	0.61
Smoking	2 (6.4)	4 (13.3)	0.37
Dyslipidemia	10 (32.3)	1 (3.3)	**0.003**
Obesity (BMI ≥ 30 kg/m^2^) (%)	8 (25.8)	5 (16.7)	0.38
Family history of hypertension (%)	18 (58.1)	6 (20.0)	**0.002**
Previous pregnancies (n)	1.7 ± 1.1	1.6 ± 1.0	0.71
Gestational age at enrollment (weeks)	32.0 ± 8.1	34.5 ± 3.8	0.13
Gestational week at delivery (weeks)	37.7 ± 1.5	39.2 ± 1.4	**<0.001**
**Hemodynamics**			
HR (bpm)	81.8 ± 12.6	78.3 ± 12.1	0.27
SBP (mmHg)	132.4 ± 14.7	110.0 ± 7.2	**<0.001**
DBP (mmHg)	85.4 ± 7.2	66.7 ± 5.8	**<0.001**
PP (mmHg)	47.1 ± 11.4	43.3 ± 7.3	0.13
MAP (mmHg)	101.1 ± 8.8	81.1 ± 5.2	**<0.001**
**Laboratory tests**			
Serum hemoglobin (g/dL)	11.6 ± 1.7	11.2 ± 1.5	0.33
eGFR (ml/min/m^2^)	138.4 ± 48.3	136.6 ± 30.1	0.86
Serum glucose (mg/dL)	83.6 ± 13.1	86.4 ± 13.8	0.42
Serum total cholesterol (mg/dL)	197.6 ± 25.5	171.2 ± 10.5	**<0.001**
Serum uric acid (mg/dL)	4.2 ± 0.4	4.1 ± 0.5	0.39
Proteinuria (%)	19 (61.3)	/	/
**Medical treatment during pregnancy**			
Calcium channel blockers (%)	11 (35.5)	/	/
Alpha2-agonists (%)	8 (25.8)	/	/
Alpha-beta blockers (%)	2 (6.5)	/	/
Dual therapy (%)	9 (29.0)	/	/
No therapy (%)	4 (12.9)	30 (100)	**<0.001**

Variables that are normally distributed are reported as mean ± standard deviation, whereas those not normally distributed are expressed as median with the range (minimum to maximum). Statistically significant *p*-values are highlighted in bold. BMI, body mass index; BSA, body surface area; DBP, diastolic blood pressure; eGFR, estimated glomerular filtration rate; HDP, hypertensive disorder of pregnancy; HR, heart rate; MAP, mean arterial pressure; PP, pulse pressure; SBP, systolic blood pressure.

**Table 3:** Clinical characteristics of the two study groups at six years postpartum.

	pHDP Women (n = 31)	Controls (n = 30)	*p*-Value
**Demographics and anthropometrics**			
Age (yrs)	42.3 ± 5.9	40.8 ± 5.0	0.29
Age ≥ 40 yrs (%)	23 (74.2)	19 (63.3)	0.36
BSA (m^2^)	1.68 ± 0.17	1.66 ± 0.14	0.62
BMI (kg/m^2^)	23.2 ± 5.2	22.2 ± 2.8	0.36
Normal weight (BMI 18.5–24.9 kg/m^2^) (%)	24 (77.4)	24 (80.0)	0.80
**Ethnicity**			
Caucasian (%)	27 (87.1)	26 (86.7)	0.96
Asiatic (%)	2 (6.5)	2 (6.7)	0.97
African (%)	1 (3.2)	1 (3.3)	0.98
Latin American (%)	1 (3.2)	1 (3.3)	0.98
**Cardiovascular risk factors**			
Smoking (%)	2 (6.5)	6 (20.0)	0.12
Type 2 diabetes mellitus (%)	2 (6.5)	1 (3.3)	0.57
Dyslipidemia (%)	7 (22.6)	1 (3.3)	**0.02**
Obesity (%)	7 (22.6)	1 (3.3)	**0.02**
**Blood pressure parameters**			
SBP (mmHg)	127.5 ± 16.8	113.2 ± 11.1	**<0.001**
DBP (mmHg)	78.4 ± 13.7	70.4 ± 9.4	**0.01**
PP (mmHg)	49.2 ± 9.9	42.8 ± 9.4	**0.01**
MAP (mmHg)	94.4 ± 13.7	84.6 ± 8.9	**<0.001**
BP ≥ 140/90 mmHg at clinical visit (%)	11 (35.5)	2 (6.7)	**0.006**
**Blood tests**			
Serum Hb (g/dL)	12.9 ± 0.8	12.7 ± 1.2	0.44
Serum creatinine (mg/dL)	0.77 ± 0.13	0.72 ± 0.16	0.18
eGFR (ml/min/m^2^)	95.2 ± 16.3	101.1 ± 18.1	0.18
Serum glucose (mg/dL)	87.1 ± 6.2	86.3 ± 7.1	0.64
Serum total cholesterol (mg/dL)	195.8 ± 13.7	192.0 ± 8.1	0.19
Serum HDL-cholesterol (mg/dL)	68.7 ± 8.2	75.2 ± 6.5	**0.001**
Serum LDL-cholesterol (mg/dL)	113.8 ± 11.4	108.2 ± 7.1	**0.02**
Serum triglycerides (mg/dL)	66.1 ± 15.4	68.7 ± 11.5	0.46
Serum uric acid (mg/dL)	4.3 ± 1.1	4.7 ± 1.4	0.22
**Comorbidities**			
Hypothyroidism (%)	4 (12.9)	8 (26.7)	0.18
**Current medical treatment**			
pHDP women in medical therapy (%)	10 (32.3)	/	/
ACE-i/ARBs (%)	6 (19.3)	/	/
Calcium channel blockers (%)	5 (16.1)	/	/
Beta blockers (%)	2 (6.5)	/	/
Diuretics (%)	1 (3.2)	/	/
Statins (%)	1 (3.2)	/	/
Thyroid hormone therapy (%)	4 (12.9)	8 (26.7)	0.18

Data with a normal distribution are presented as mean ± standard deviation, whereas data that are not normally distributed are shown as median along with the range (minimum to maximum). *p*-values considered statistically significant are highlighted in bold. ACE-i, angiotensin-converting enzyme inhibitors; ARBs, angiotensin II receptor blockers; BMI, body mass index; BP, blood pressure; BSA, body surface area; DBP, diastolic blood pressure; eGFR, estimated glomerular filtration rate; HDL, high-density lipoprotein; HDP, hypertensive disorder of pregnancy; LDL, low-density lipoprotein; MAP, mean arterial pressure; PP, pulse pressure; SBP, systolic blood pressure.

**Table 4:** Morphological, functional, and hemodynamic parameters assessed by conventional transthoracic echocardiography and carotid ultrasonography in the two groups of women at six years postpartum.

	pHDP Women (n = 31)	Controls (n = 30)	*p*-Value
**Yrs postpartum at echocardiographic assessment**	6.1 ± 1.3	6.0 ± 0.3	0.68
**Conventional echoDoppler parameters**			
IVS (mm)	9.4 ± 1.7	7.6 ± 1.2	**<0.001**
LV-PW (mm)	7.4 ± 1.1	6.6 ± 1.0	**0.004**
LV-EDD (mm)	42.6 ± 3.8	44.4 ± 2.7	**0.04**
RWT	0.35 ± 0.05	0.30 ± 0.05	**<0.001**
LVMi (g/m^2^)	66.6 ± 13.9	57.7 ± 9.7	**0.005**
Normal LV geometric pattern (%)	27 (87.1)	28 (93.4)	0.41
LV concentric remodeling (%)	3 (9.7)	1 (3.3)	0.32
LV eccentric remodeling (%)	1 (3.2)	1 (3.3)	0.98
LVEDVi (ml/m^2^)	35.7 ± 6.6	35.3 ± 5.6	0.79
LVESVi (ml/m^2^)	11.6 ± 2.4	11.9 ± 2.5	0.63
LVEF (%)	66.9 ± 3.1	65.9 ± 4.8	0.33
E/A ratio	1.14 (0.67–1.76)	1.34 (0.71–2.1)	**0.02**
E/e’ ratio	8.02 ± 2.32	5.14 ± 1.34	**<0.001**
LA A-P diameter (mm)	34.3 ± 5.4	33.6 ± 4.1	0.57
LA longitudinal diameter (mm)	44.8 ± 6.1	46.4 ± 4.9	0.26
LAVi (ml/m^2^)	29.0 ± 7.5	27.4 ± 7.3	0.40
Mild MR (n, %)	12 (38.7)	9 (30.0)	0.95
Mild TR (n, %)	19 (61.3)	17 (56.6)	0.71
RVIT (mm)	28.9 (24–36)	29.7 (23.5–34)	0.31
TAPSE (mm)	24.6 (20–30)	26.4 (19–32)	**0.04**
IVC (mm)	15.2 ± 4.1	17.0 ± 3.9	0.08
sPAP (mmHg)	23.9 ± 3.1	22.8 ± 2.2	0.12
TAPSE/sPAP ratio	1.05 ± 0.18	1.17 ± 0.18	**0.01**
Aortic root (mm)	30.8 ± 2.6	29.1 ± 2.6	**0.01**
Ascending aorta (mm)	29.5 ± 4.1	28.8 ± 3.1	0.46
**Hemodynamic indices**			
Heart rate (bpm)	79.6 (58–103)	75.5 (62–100)	0.19
ESP (mmHg)	114.1 ± 14.8	101.9 ± 10.0	**<0.001**
SVi (mL/m^2^)	35.2 ± 6.9	39.5 ± 9.1	**0.04**
COi (L/min/m^2^)	2.81 ± 0.74	2.93 ± 0.67	0.51
TPRi (dyne.sec/cm^5^)/m^2^	2863.3 ± 837.6	2427.5 ± 620.6	**0.02**
EaI (mmHg/mL/m^2^)	3.4 ± 0.9	2.7 ± 0.7	**0.001**
EesI (mmHg/mL/m^2^)	10.1 ± 2.1	9.0 ± 2.4	0.06
EaI/EesI ratio	0.34 ± 0.10	0.32 ± 0.09	0.41
**Carotid parameters**			
Av. CCA-EDD (mm)	6.64 ± 0.53	6.64 ± 0.44	>0.99
Av. CCA-IMT (mm)	0.90 ± 0.21	0.62 ± 0.19	**<0.001**
Av. CCA-IMT ≥ 0.7 mm (%)	27 (87.1)	7 (23.3)	**<0.001**
Av. CCA-RWT	0.28 ± 0.08	0.19 ± 0.06	**<0.001**
Av. CCA-CSA (mm^2^)	22.90 ± 7.91	14.20 ± 4.94	**<0.001**

Variables that follow a normal distribution are reported as the mean ± standard deviation, whereas non-normally distributed data are presented as the median along with the range (minimum to maximum). Statistically significant *p*-values are highlighted in bold. A-P, antero-posterior; CCA, common carotid artery; COi, cardiac output indexed; CSA, cross-sectional area; EaI, arterial elastance indexed; EDD, end-diastolic diameter; EesI, end-systolic elastance indexed; ESP, end-systolic pressure; IMT, intima-media thickness; IVC, inferior vena cava; IVS, interventricular septum; LA, left atrial; LAVi, left atrial volume indexed; LV, left ventricular; LVEDD, left ventricular end-diastolic diameter; LVEDVi, left ventricular end-diastolic volume indexed; LVESVi, left ventricular end-systolic volume indexed; MR, mitral regurgitation; PW, posterior wall; RVIT, right ventricular inflow tract; RWT, relative wall thickness; sPAP, systolic pulmonary artery pressure; SVi, stroke volume indexed; TAPSE, tricuspid annular plane systolic excursion; TPRi, total peripheral resistance index; TR, tricuspid regurgitation.

**Table 5:** Biventricular and biatrial strain parameters measured by speckle tracking echocardiography in the two study groups at six years postpartum.

STE Variables	pHDP Women (n = 31)	Controls (n = 30)	*p*-Value
LV-GLS (%)	19.5 ± 2.6	22.3 ± 2.3	**<0.001**
LV-GLSR (s^−1^)	1.1 ± 0.1	1.2 ± 0.1	**<0.001**
LV-GCS (%)	24.5 ± 5.5	26.7 ± 4.4	0.09
LV-GCSR (s^−1^)	1.6 ± 0.3	1.7 ± 0.2	0.13
LAScd (%)	30.1 ± 7.3	36.3 ± 7.7	**0.002**
LASct (%)	7.7 ± 4.8	9.4 ± 4.1	0.14
LASr (%)	37.8 ± 8.0	45.7 ± 8.0	**<0.001**
LASr/E/e’	5.1 ± 1.8	9.5 ± 3.2	**<0.001**
LA-GSR+ (s^−1^)	2.0 ± 0.5	2.3 ± 0.5	**0.02**
LA-GSRE (s^−1^)	2.4 ± 0.8	3.1 ± 0.8	**0.001**
LA-GSRL (s^−1^)	2.7 ± 0.7	2.8 ± 0.5	0.52
RV-FWLS (%)	19.8 ± 3.6	22.0 ± 3.5	**0.02**
RV-GLS (%)	18.6 ± 3.3	20.9 ± 3.4	**0.01**
RV-GLSR (s^−1^)	1.2 ± 0.2	1.3 ± 0.2	0.06
RAScd (%)	28.0 ± 8.2	34.6 ± 10.1	**0.007**
RASct (%)	7.2 ± 4.7	7.5 ± 5.4	0.82
RASr (%)	35.2 ± 7.7	42.1 ± 9.9	**0.004**
RA-GSR+ (s^−1^)	2.2 ± 0.5	2.4 ± 0.6	0.16
RA-GSRE (s^−1^)	1.9 (1.1–3.0)	2.3 (1.3–3.5)	**0.02**
RA-GSRL (s^−1^)	2.3 (1.1–4.0)	2.5 (1.3–5.0)	0.30
**Percentage of Women with Impaired STE Parameters Compared to Reference Values**			
LV-GLS < 20% (%)	18 (58.1)	4 (13.3)	**<0.001**
LV-GCS < 23.3% (%)	12 (38.7)	7 (23.3)	0.19
LASr < 39% (%)	17 (54.8)	5 (16.7)	**0.002**
RV-GLS < 20% (%)	22 (71.0)	11 (36.7)	**0.007**
RASr < 35% (%)	18 (58.1)	8 (26.7)	**0.01**

Variables with a normal distribution are presented as mean ± standard deviation, whereas variables that are not normally distributed are reported as median along with the range (minimum to maximum). *p*-values indicating statistical significance are highlighted in bold. FWLS, free wall longitudinal strain; GCS, global circumferential strain; GCSR, global circumferential strain rate; GLS, global longitudinal strain; GLSR, global longitudinal strain rate; GSR+, positive global strain rate; GSRE, global early-diastolic strain rate; GSRL, global late-diastolic strain rate; LAScd, left atrial conduit strain; LASct, left atrial contractile strain; LASr, left atrial reservoir strain; LV, left ventricular; RAScd, right atrial conduit strain; RASct, right atrial contractile strain; RASr, right atrial reservoir strain; RV, right ventricular; STE, speckle tracking echocardiography.

**Table 6:** Univariate and multivariate Cox regression analyses conducted to identify independent predictors of subclinical myocardial dysfunction at six years postpartum. Significant *p*-values are in bold. BMI, body mass index; PE, pre-eclampsia.

	Univariate Cox Regression Analysis			Multivariate Cox Regression Analysis		
Variables	HR	95% CI	*p*-Value	HR	95% CI	*p*-Value
Third-trimester age (yrs)	1.00	0.89–1.06	0.47			
Third-trimester BMI (kg/m^2^)	1.12	1.02–1.22	**0.02**	1.05	0.95–1.16	0.36
Previous PE	5.09	1.47–17.6	**0.01**	4.01	1.05–15.3	**0.03**
Chronic antihypertensive treatment	0.97	0.34–2.77	0.96			

**Table 7:** Univariate and multivariate Cox regression analyses carried out to determine the independent factors predicting subclinical carotid atherosclerosis at six years postpartum. Statistically significant *p*-values are highlighted in bold. BMI, body mass index; HDL, high-density lipoprotein; PE, pre-eclampsia.

	Univariate Cox Regression Analysis			Multivariate Cox Regression Analysis		
Variables	HR	95% CI	*p*-Value	HR	95% CI	*p*-Value
Third-trimester age (yrs)	1.01	0.93–1.09	0.86			
Third-trimester BMI (kg/m^2^)	1.14	1.04–1.24	**0.004**	1.21	1.07–1.38	**0.003**
Previous PE	4.49	1.31–15.4	**0.02**	6.38	1.50–27.2	**0.01**
Current HDL-cholesterol (mg/dl)	0.99	0.94–1.05	0.86			

## Data Availability

Data extracted from included studies will be publicly available on Zenodo (https://zenodo.org) (accessed on 7 June 2025).

## References

[1] (2019). Hypertension in Pregnancy: Diagnosis and Management.

[2] Regitz-Zagrosek V., Roos-Hesselink J.W., Bauersachs J., Blomström-Lundqvist C., Cífková R., De Bonis M., Iung B., Johnson M.R., Kintscher U., Kranke P. (2018). 2018 ESC Guidelines for the management of cardiovascular diseases during pregnancy. Eur. Heart J..

[3] (2020). Gestational Hypertension and Preeclampsia: ACOG Practice Bulletin; Number 222. Obstet. Gynecol..

[4] Maas A.H.E.M., Rosano G., Cifkova R., Chieffo A., van Dijken D., Hamoda H., Kunadian V., Laan E., Lambrinoudaki I., Maclaran K. (2021). Cardiovascular health after menopause transition; pregnancy disorders; and other gynaecologic conditions: A consensus document from European cardiologists; gynaecologists; and endocrinologists. Eur. Heart J..

[5] Grandi S.M., Filion K.B., Yoon S., Ayele H.T., Doyle C.M., Hutcheon J.A., Smith G.N., Gore G.C., Ray J.G., Nerenberg K. (2019). Cardiovascular Disease-Related Morbidity and Mortality in Women with a History of Pregnancy Complications. Circulation.

[6] Wang Y.X., Arvizu M., Rich-Edwards J.W., Wang L., Rosner B., Stuart J.J., Rexrode K.M., Chavarro J.E. (2021). Hypertensive Disorders of Pregnancy and Subsequent Risk of Premature Mortality. J. Am. Coll. Cardiol..

[7] Ying W., Catov J.M., Ouyang P. (2018). Hypertensive Disorders of Pregnancy and Future Maternal Cardiovascular Risk. J. Am. Heart Assoc..

[8] Kestenbaum B., Seliger S.L., Easterling T.R., Gillen D.L., Critchlow C.W., Stehman-Breen C.O., Schwartz S.M. (2003). Cardiovascular and thromboembolic events following hypertensive pregnancy. Am. J. Kidney Dis..

[9] Cain M.A., Salemi J.L., Tanner J.P., Kirby R.S., Salihu H.M., Louis J.M. (2016). Pregnancy as a window to future health: Maternal placental syndromes and short-term cardiovascular outcomes. Am. J. Obstet. Gynecol..

[10] Egeland G.M., Skurtveit S., Staff A.C., Eide G.E., Daltveit A.K., Klungsøyr K., Trogstad L., Magnus P.M., Brantsæter A.L., Haugen M. (2018). Pregnancy-Related Risk Factors Are Associated with a Significant Burden of Treated Hypertension Within 10 Years of Delivery: Findings from a Population-Based Norwegian Cohort. J. Am. Heart Assoc..

[11] Jarvie J.L., Metz T.D., Davis M.B., Ehrig J.C., Kao D.P. (2018). Short-term risk of cardiovascular readmission following a hypertensive disorder of pregnancy. Heart.

[12] Luis S.A., Chan J., Pellikka P.A. (2019). Echocardiographic Assessment of Left Ventricular Systolic Function: An Overview of Contemporary Techniques; Including Speckle-Tracking Echocardiography. Mayo Clin. Proc..

[13] Voigt J.U., Cvijic M. (2019). 2- and 3-Dimensional Myocardial Strain in Cardiac Health and Disease. JACC Cardiovasc. Imaging.

[14] Clemmensen T.S., Christensen M., Kronborg C.J.S., Knudsen U.B., Løgstrup B.B. (2018). Long-term follow-up of women with early onset pre-eclampsia shows subclinical impairment of the left ventricular function by two-dimensional speckle tracking echocardiography. Pregnancy Hypertens..

[15] Boardman H., Lamata P., Lazdam M., Verburg A., Siepmann T., Upton R., Bilderbeck A., Dore R., Smedley C., Kenworthy Y. (2020). Variations in Cardiovascular Structure; Function; and Geometry in Midlife Associated with a History of Hypertensive Pregnancy. Hypertension.

[16] Levine L.D., Ky B., Chirinos J.A., Koshinksi J., Arany Z., Riis V., Elovitz M.A., Koelper N., Lewey J. (2022). Prospective Evaluation of Cardiovascular Risk 10 Years After a Hypertensive Disorder of Pregnancy. J. Am. Coll. Cardiol..

[17] Gronningsaeter L., Skulstad H., Quattrone A., Langesaeter E., Estensen M.E. (2022). Reduced left ventricular function and sustained hypertension in women seven years after severe preeclampsia. Scand. Cardiovasc. J..

[18] Al-Nashi M., Eriksson M.J., Östlund E., Bremme K., Kahan T. (2016). Cardiac structure and function; and ventricular-arterial interaction 11 years following a pregnancy with preeclampsia. J. Am. Soc. Hypertens..

[19] Sonaglioni A., Lonati C., Lombardo M., Rigamonti E., Binda G., Vincenti A., Nicolosi G.L., Bianchi S., Harari S., Anzà C. (2019). Incremental prognostic value of global left atrial peak strain in women with new-onset gestational hypertension. J. Hypertens..

[20] Sjaus A., McKeen D.M., George R.B. (2016). Hypertensive disorders of pregnancy. Can. J. Anaesth..

[21] Magee L.A., Pels A., Helewa M., Rey E., von Dadelszen P., Canadian Hypertensive Disorders of Pregnancy (HDP) Working Group (2014). Diagnosis; evaluation; and management of the hypertensive disorders of pregnancy. Pregnancy Hypertens..

[22] Casiglia E. (2024). AND, OR, AND/OR in hypertension guidelines. J. Hypertens..

[23] Levey A.S., Bosch J.P., Lewis J.B., Greene T., Rogers N., Roth D. (1999). A more accurate method to estimate glomerular filtration rate from serum creatinine: A new prediction equation. Modification of Diet in Renal Disease Study Group. Ann. Intern. Med..

[24] Lang R.M., Badano L.P., Mor-Avi V., Afilalo J., Armstrong A., Ernande L., Flachskampf F.A., Foster E., Goldstein S.A., Kuznetsova T. (2015). Recommendations for cardiac chamber quantification by echocardiography in adults: An update from the American Society of Echocardiography and the European Association of Cardiovascular Imaging. J. Am. Soc. Echocardiogr..

[25] Nagueh S.F., Smiseth O.A., Appleton C.P., Byrd B.F., Dokainish H., Edvardsen T., Flachskampf F.A., Gillebert T.C., Klein A.L., Lancellotti P. (2016). Recommendations for the Evaluation of Left Ventricular Diastolic Function by Echocardiography: An Update from the American Society of Echocardiography and the European Association of Cardiovascular Imaging. J. Am. Soc. Echocardiogr..

[26] Humbert M., Kovacs G., Hoeper M.M., Badagliacca R., Berger R.M.F., Brida M., Carlsen J., Coats A.J.S., Escribano-Subias P., Ferrari P. (2023). 2022 ESC/ERS Guidelines for the diagnosis and treatment of pulmonary hypertension. Eur. Respir. J..

[27] Tello K., Wan J., Dalmer A., Vanderpool R., Ghofrani H.A., Naeije R., Roller F., Mohajerani E., Seeger W., Herberg U. (2019). Validation of the Tricuspid Annular Plane Systolic Excursion/Systolic Pulmonary Artery Pressure Ratio for the Assessment of Right Ventricular-Arterial Coupling in Severe Pulmonary Hypertension. Circ. Cardiovasc. Imaging.

[28] Zheng L., Sun Z., Li J., Zhang R., Zhang X., Liu S., Li J., Xu C., Hu D., Sun Y. (2008). Pulse pressure and mean arterial pressure in relation to ischemic stroke among patients with uncontrolled hypertension in rural areas of China. Stroke.

[29] Franklin S.S., Wong N.D. (2016). Pulse Pressure: How Valuable as a Diagnostic and Therapeutic Tool?. J. Am. Coll. Cardiol..

[30] Sattin M., Burhani Z., Jaidka A., Millington S.J., Arntfield R.T. (2022). Stroke Volume Determination by Echocardiography. Chest.

[31] Hill L.K., Sollers Iii J.J., Thayer J.F. (2013). Resistance reconstructed estimation of total peripheral resistance from computationally derived cardiac output—Biomed. Biomed. Sci. Instrum..

[32] Redfield M.M., Jacobsen S.J., Borlaug B.A., Rodeheffer R.J., Kass D.A. (2005). Age- and gender-related ventricular-vascular stiffening: A community-based study. Circulation.

[33] Chantler P.D., Lakatta E.G., Najjar S.S. (2008). Arterial-ventricular coupling: Mechanistic insights into cardiovascular performance at rest and during exercise. J. Appl. Physiol. (1985).

[34] Voigt J.U., Pedrizzetti G., Lysyansky P., Marwick T.H., Houle H., Baumann R., Pedri S., Ito Y., Abe Y., Metz S. (2015). Definitions for a common standard for 2D speckle tracking echocardiography: Consensus document of the EACVI/ASE/Industry Task Force to standardize deformation imaging. Eur. Heart J. Cardiovasc. Imaging.

[35] Espersen C., Skaarup K.G., Lassen M.C.H., Johansen N.D., Hauser R., Jensen G.B., Schnohr P., Møgelvang R., Biering-Sørensen T. (2024). Right ventricular free wall and four-chamber longitudinal strain in relation to incident heart failure in the general population. Eur. Heart J. Cardiovasc. Imaging.

[36] Voigt J.U., Mălăescu G.G., Haugaa K., Badano L. (2020). How to do LA strain. Eur. Heart J. Cardiovasc. Imaging.

[37] Sonaglioni A., Vincenti A., Baravelli M., Rigamonti E., Tagliabue E., Bassi P., Nicolosi G.L., Anzà C., Lombardo M. (2019). Prognostic value of global left atrial peak strain in patients with acute ischemic stroke and no evidence of atrial fibrillation. Int. J. Cardiovasc. Imaging.

[38] Galderisi M., Cosyns B., Edvardsen T., Cardim N., Delgado V., Di Salvo G., Donal E., Sade L.E., Ernande L., Garbi M. (2017). Standardization of adult transthoracic echocardiography reporting in agreement with recent chamber quantification; diastolic function; and heart valve disease recommendations: An expert consensus document of the European Association of Cardiovascular Imaging. Eur. Heart J. Cardiovasc. Imaging.

[39] Yingchoncharoen T., Agarwal S., Popović Z.B., Marwick T.H. (2013). Normal ranges of left ventricular strain: A meta-analysis. J. Am. Soc. Echocardiogr..

[40] Muraru D., Onciul S., Peluso D., Soriani N., Cucchini U., Aruta P., Romeo G., Cavalli G., Iliceto S., Badano L.P. (2016). Sex- and Method-Specific Reference Values for Right Ventricular Strain by 2-Dimensional Speckle-Tracking Echocardiography. Circ. Cardiovasc. Imaging.

[41] Pathan F., D’Elia N., Nolan M.T., Marwick T.H., Negishi K. (2017). Normal Ranges of Left Atrial Strain by Speckle-Tracking Echocardiography: A Systematic Review and Meta-Analysis. J. Am. Soc. Echocardiogr..

[42] Krittanawong C., Maitra N.S., Hassan Virk H.U., Farrell A., Hamzeh I., Arya B., Pressman G.S., Wang Z., Marwick T.H. (2023). Normal Ranges of Right Atrial Strain: A Systematic Review and Meta-Analysis. JACC Cardiovasc. Imaging.

[43] Stein J.H., Korcarz C.E., Hurst R.T., Lonn E., Kendall C.B., Mohler E.R., Najjar S.S., Rembold C.M., Post W.S., American Society of Echocardiography Carotid Intima-Media Thickness Task Force (2008). Use of carotid ultrasound to identify subclinical vascular disease and evaluate cardiovascular disease risk: A consensus statement from the American Society of Echocardiography Carotid Intima-Media Thickness Task Force. Endorsed by the Society for Vascular Medicine. J. Am. Soc. Echocardiogr..

[44] Lorenz M.W., von Kegler S., Steinmetz H., Markus H.S., Sitzer M. (2006). Carotid intima-media thickening indicates a higher vascular risk across a wide age range: Prospective data from the Carotid Atherosclerosis Progression Study (CAPS). Stroke.

[45] Randrianarisoa E., Rietig R., Jacob S., Blumenstock G., Haering H.U., Rittig K., Balletshofer B. (2015). Normal values for intima-media thickness of the common carotid artery—An update following a novel risk factor profiling. Vasa.

[46] Holm H., Magnusson M., Jujić A., Pugliese N.R., Bozec E., Lamiral Z., Huttin O., Zannad F., Rossignol P., Girerd N. (2023). Ventricular-arterial coupling (VAC) in a population-based cohort of middle-aged individuals: The STANISLAS cohort. Atherosclerosis.

[47] Galderisi M., Lomoriello V.S., Santoro A., Esposito R., Olibet M., Raia R., Di Minno M.N., Guerra G., Mele D., Lombardi G. (2010). Differences of myocardial systolic deformation and correlates of diastolic function in competitive rowers and young hypertensives: A speckle-tracking echocardiography study. J. Am. Soc. Echocardiogr..

[48] Kornev M., Caglayan H.A., Kudryavtsev A.V., Malyutina S., Ryabikov A., Schirmer H., Rösner A. (2023). Influence of hypertension on systolic and diastolic left ventricular function including segmental strain and strain rate. Echocardiography.

[49] Ikonomidis I., Aboyans V., Blacher J., Brodmann M., Brutsaert D.L., Chirinos J.A., De Carlo M., Delgado V., Lancellotti P., Lekakis J. (2019). The role of ventricular-arterial coupling in cardiac disease and heart failure: Assessment; clinical implications and therapeutic interventions. A consensus document of the European Society of Cardiology Working Group on Aorta & Peripheral Vascular Diseases; European Association of Cardiovascular Imaging; and Heart Failure Association. Eur. J. Heart Fail..

[50] He Q., Lin Y., Zhu Y., Gao L., Ji M., Zhang L., Xie M., Li Y. (2023). Clinical Usefulness of Right Ventricle-Pulmonary Artery Coupling in Cardiovascular Disease. J. Clin. Med..

[51] Cañon-Montañez W., Santos A.B.S., Nunes L.A., Pires J.C.G., Freire C.M.V., Ribeiro A.L.P., Mill J.G., Bessel M., Duncan B.B., Schmidt M.I. (2018). Central Obesity is the Key Component in the Association of Metabolic Syndrome with Left Ventricular Global Longitudinal Strain Impairment. Rev. Esp. Cardiol. (Engl. Ed.).

[52] Sawada N., Nakanishi K., Daimon M., Yoshida Y., Ishiwata J., Hirokawa M., Nakao T., Morita H., Di Tullio M.R., Homma S. (2020). Influence of visceral adiposity accumulation on adverse left and right ventricular mechanics in the community. Eur. J. Prev. Cardiol..

[53] Sonaglioni A., Esposito V., Caruso C., Nicolosi G.L., Bianchi S., Lombardo M., Gensini G.F., Ambrosio G. (2021). Chest conformation spuriously influences strain parameters of myocardial contractile function in healthy pregnant women. J. Cardiovasc. Med..

[54] Sonaglioni A., Ferrulli A., Nicolosi G.L., Lombardo M., Luzi L. (2024). The Influence of Anthropometrics on Cardiac Mechanics in Healthy Women with Opposite Obesity Phenotypes (Android vs Gynoid). Cureus.

[55] Sonaglioni A., Fagiani V., Nicolosi G.L., Lombardo M. (2024). Echocardiographic assessment of left ventricular mechanics in individuals with mitral valve prolapse: A systematic review and meta-analysis. Int. J. Cardiovasc. Imaging.

[56] Andersgaard A.B., Acharya G., Mathiesen E.B., Johnsen S.H., Straume B., Øian P. (2012). Recurrence and long-term maternal health risks of hypertensive disorders of pregnancy: A population-based study. Am. J. Obstet. Gynecol..

[57] Goynumer G., Yucel N., Adali E., Tan T., Baskent E., Karadag C. (2013). Vascular risk in women with a history of severe preeclampsia. J. Clin. Ultrasound..

[58] Aykas F., Solak Y., Erden A., Bulut K., Dogan S., Sarli B., Acmaz G., Afsar B., Siriopol D., Covic A. (2015). Persistence of cardiovascular risk factors in women with previous preeclampsia: A long-term follow-up study. J. Investig. Med..

[59] Christensen M., Kronborg C.S., Carlsen R.K., Eldrup N., Knudsen U.B. (2017). Early gestational age at preeclampsia onset is associated with subclinical atherosclerosis 12 years after delivery. Acta Obstet. Gynecol. Scand..

[60] Garrido-Gimenez C., Mendoza M., Cruz-Lemini M., Galian-Gay L., Sanchez-Garcia O., Granato C., Rodriguez-Sureda V., Rodriguez-Palomares J., Carreras-Moratonas E., Cabero-Roura L. (2020). Angiogenic Factors and Long-Term Cardiovascular Risk in Women That Developed Preeclampsia During Pregnancy. Hypertension.

[61] Amor A.J., Vinagre I., Valverde M., Alonso N., Urquizu X., Meler E., López E., Giménez M., Codina L., Conget I. (2021). Novel glycoproteins identify preclinical atherosclerosis among women with previous preeclampsia regardless of type 1 diabetes status. Nutr. Metab. Cardiovasc. Dis..

[62] Fraser A., Nelson S.M., Macdonald-Wallis C., Cherry L., Butler E., Sattar N., Lawlor D.A. (2012). Associations of pregnancy complications with calculated cardiovascular disease risk and cardiovascular risk factors in middle age: The Avon Longitudinal Study of Parents and Children. Circulation.

[63] Romundstad P.R., Magnussen E.B., Smith G.D., Vatten L.J. (2010). Hypertension in pregnancy and later cardiovascular risk: Common antecedents?. Circulation.

[64] Staff A.C., Johnsen G.M., Dechend R., Redman C.W.G. (2014). Preeclampsia and uteroplacental acute atherosis: Immune and inflammatory factors. J. Reprod. Immunol..

[65] Veerbeek J.H., Brouwers L., Koster M.P., Koenen S.V., van Vliet E.O., Nikkels P.G., Franx A., van Rijn B.B. (2016). Spiral artery remodeling and maternal cardiovascular risk: The spiral artery remodeling (SPAR) study. J. Hypertens..

[66] Agatisa P.K., Ness R.B., Roberts J.M., Costantino J.P., Kuller L.H., McLaughlin M.K. (2004). Impairment of endothelial function in women with a history of preeclampsia: An indicator of cardiovascular risk. Am. J. Physiol. Heart Circ. Physiol..

[67] Kvehaugen A.S., Dechend R., Ramstad H.B., Troisi R., Fugelseth D., Staff A.C. (2011). Endothelial function and circulating biomarkers are disturbed in women and children after preeclampsia. Hypertension.

[68] American College of Obstetricians and Gynecologists’ Committee on Practice Bulletins—Obstetrics (2019). ACOG Practice Bulletin No. 203: Chronic Hypertension in Pregnancy. Obstet. Gynecol..

[69] Mureddu G.F. (2023). How much does hypertension in pregnancy affect the risk of future cardiovascular events?. Eur. Heart J. Suppl..

[70] Negishi T., Negishi K., Thavendiranathan P., Cho G.Y., Popescu B.A., Vinereanu D., Kurosawa K., Penicka M., Marwick T.H., SUCCOUR Investigators (2017). Effect of Experience and Training on the Concordance and Precision of Strain Measurements. JACC Cardiovasc. Imaging.

[71] Rösner A., Barbosa D., Aarsæther E., Kjønås D., Schirmer H., D’hooge J. (2015). The influence of frame rate on two-dimensional speckle-tracking strain measurements: A study on silico-simulated models and images recorded in patients. Eur. Heart J. Cardiovasc. Imaging.

[72] Mirea O., Pagourelias E.D., Duchenne J., Bogaert J., Thomas J.D., Badano L.P., Voigt J.U., EACVI-ASE-Industry Standardization Task Force (2018). Intervendor Differences in the Accuracy of Detecting Regional Functional Abnormalities: A Report From the EACVI-ASE Strain Standardization Task Force. JACC Cardiovasc. Imaging.

[73] Sonaglioni A., Fagiani V., Nicolosi G.L., Lombardo M. (2024). The influence of pectus excavatum on biventricular mechanics: A systematic review and meta-analysis. Minerva Cardiol. Angiol..

